# Application of Graphene Oxide for Adsorption Removal of Geosmin and 2-Methylisoborneol in the Presence of Natural Organic Matter

**DOI:** 10.3390/ijerph16111907

**Published:** 2019-05-30

**Authors:** Akira Hafuka, Takahiro Nagasato, Hiroshi Yamamura

**Affiliations:** 1 Center for Regional Environmental Research, National Institute for Environmental Studies (NIES), 16-2 Onogawa, Tsukuba, Ibaraki 305-8506, Japan; 2 Department of Integrated Science and Engineering for Sustainable Society, Faculty of Science and Engineering, Chuo University, 1-13-27 Kasuga, Bunkyo-ku, Tokyo 112-8551, Japan; tn060613@gmail.com (T.N.); yamamura.10x@g.chuo-u.ac.jp (H.Y.)

**Keywords:** taste and odor, drinking water treatment, BET isotherm, Langmuir isotherm

## Abstract

We investigated the adsorption characteristics of geosmin and 2-methylisoborneol (MIB) on graphene oxide (GO) in the absence and presence of natural organic matter (NOM). The graphene oxide had fast adsorption kinetics for both compounds because of its open-layered structure, with adsorption equilibrium being achieved within 15 min of contact. Although NOM did not affect the adsorption of geosmin on GO, it delayed that of MIB, probably because of competition for adsorption sites. The adsorption isotherms show that GO had a greater capacity for geosmin adsorption than for MIB because geosmin was more hydrophobic. Moreover, NOM interfered with the adsorption of MIB onto the GO, but increased the amount of adsorbed geosmin, which likely occurred because NOM increased the dispersibility of GO, which then increased the number of GO adsorption sites. The difference in the effects of NOM on GO adsorption of geosmin and MIB may be explained by their hydrophobicity. Although the adsorption of geosmin and MIB by GO was fast, its capacity to adsorb both compounds was substantially lower than that of activated carbon because of its higher hydrophilicity.

## 1. Introduction

Geosmin and 2-methylisoborneol (MIB) are musty-earthy odor compounds that are commonly found in surface water after being produced as metabolites by various microorganisms, such as cyanobacteria [1]. Their chemical structures and other properties are shown in Table 1. Both compounds are found in raw water subjected to drinking water treatment and are sometimes present in tap water from public drinking water supplies. It is necessary to remove these compounds during drinking water treatment because they lead to customer complaints, even at low concentration (10 ng/L). In Japan, the drinking water quality standards for geosmin and MIB are both 10 ng/L. Ozone oxidation and adsorption with granular or powdered activated carbon (PAC) are used to remove micropollutants, including geosmin and MIB [2]. Adsorption is simpler than ozone oxidation, and PAC is widely used in drinking water treatment plants. The adsorption capacity of PAC for both geosmin and MIB is high because of its surface hydrophobicity and large specific surface area. However, the adsorption kinetics are very slow; therefore, long contact times (3–5 d) are needed to reach adsorption equilibrium [3]. To overcome this disadvantage, superfine powdered activated carbon (SPAC) with a particle diameter <1 μm has been developed [4,5]. Although the adsorbate uptake rate has been improved in SPAC because of the shortened diffusion distance within SPAC particles, the kinetics are still slow and one week is needed to reach adsorption equilibrium because of its improved adsorption capacity [5,6].

Graphene oxide (GO) is a novel carbon nanomaterial derived from graphene that consists of a single-layer sheet structure with a thickness of one carbon atom and a two-dimensional honeycomb structure with sp^2^-hybridized carbon atoms [7]. Graphene oxide possesses several oxygen-containing functional groups, including epoxides, hydroxy groups, and carboxyl groups on its surface and at its edges [8]. Therefore, GO has a high hydrophilicity and is well dispersed in water. Because of its interesting physicochemical properties, GO has been widely investigated in the field of electronics, sensors, composite materials, and biomedical applications [9,10,11,12]. Recently, GO has been used as an adsorbent for organic pollutants such as dyes, polycyclic aromatic hydrocarbons, pesticides, pharmaceuticals, and personal care products [13,14,15,16]. However, its application for the adsorption removal of geosmin and MIB has not been reported. In previous studies, the average specific surface area of GO was reported to be 624 m^2^/g, indicating it will have high adsorption capacities for organic pollutants [13]. In addition, rapid adsorption kinetics can be achieved because there is no diffusion of pollutants owing to the open-layered structure of GO. Geosmin and MIB always coexist with natural organic matter (NOM) in raw water for drinking water treatment. Therefore, NOM may show interfering effects on the adsorption removal of geosmin and MIB. Thus, in this study, we applied GO for the adsorption removal of geosmin and MIB, and then investigated the effects of NOM on the adsorption of these odor compounds by GO.

## 2. Materials and Methods

### 2.1. Chemicals

Graphene oxide dispersion in water (4 mg/mL) was purchased from Sigma-Aldrich (product number: 777676, St. Louis, MO, USA) and used as an adsorbent. Geosmin and MIB standards were purchased from Wako Pure Chemical Industries (Osaka, Japan). International Humic Substances Society Suwannee River NOM (RO isolation, 2R101N) was used as the NOM substance standard. A mixed standard solution of geosmin and MIB in methanol was purchased from Wako Pure Chemical Industries and used to generate a calibration curve. Standard solution of 2,4,6-trichloroanisole-d_3_ in methanol was also purchased from Wako Pure Chemical Industries and used as an internal standard to generate a calibration curve. All chemicals were used without further purification.

### 2.2. Water Samples and Testing

Stock solutions of geosmin and MIB (~100 mg/L each) were prepared by dissolving pure geosmin or MIB standard in Milli-Q water (18.25 MΩcm) and then filtering the solution through a membrane with 0.2 μm pore size (T020A047A; Toyo Roshi Kaisya, Ltd., Tokyo, Japan). The NOM stock solution (43.9 mg C/L) was prepared by dissolving the NOM standard in Milli-Q water and then filtering the solution through a 0.2 μm pore size membrane. The dissolved organic carbon concentration of NOM stock solution was determined using a total organic carbon analyzer (TOC-L CSH; Shimadzu Corporation, Kyoto, Japan). The phosphate buffer solution (500 mM, pH 6.9) was prepared by dissolving NaH_2_PO_4_ and Na_2_HPO_4_ in Milli-Q water. For the adsorption experiments, water samples were prepared by adding stock solutions and phosphate buffer solution into Milli-Q water. The initial concentrations of geosmin and MIB were ~1 μg/L, while the NOM was ~3 mg C/L, and the phosphate buffer concentration of the samples was 5 mM. Concentrations of geosmin and MIB were determined using a headspace sampler (HS-20; Shimadzu Corp., Kyoto, Japan) coupled to a gas chromatograph-mass spectrometer (GCMS-QP2010 Ultra; Shimadzu Corp., Kyoto, Japan) equipped with a Rtx-5MS capillary column (GL Sciences Inc., Tokyo, Japan) after 2,4,6-trichloroanisole-d_3_ was added as an internal standard. The target *m/z* values of geosmin and MIB were 95 and 112, respectively, and the target *m/z* of 2,4,6-trichloroanisole-d_3_ was 213.

### 2.3. Batch Adsorption Experiments

In the adsorption kinetic experiments, 100 mL aliquots of water samples were added into 110-mL vials, after which the GO dispersions were added (100 mg/L). The vials were closed with PTFE caps, manually shaken, and then mechanically stirred at room temperature. Control samples without GO were also prepared. During the adsorption experiments, 10 mL of the solution was collected from the vials and centrifuged at 10,000 rpm for 3 min. The supernatant was subsequently filtered through a 0.45 μm pore size membrane (T020A047A; Toyo Roshi Kaisya, Ltd., Tokyo, Japan), after which the filtrate was diluted by 10×, then 10 mL of the solution and 3.5 g of NaCl were added into a 20 mL vial. Next, the concentrations of geosmin and MIB in the prepared samples were determined by headspace GC-MS after 2,4,6-trichloroanisole-d_3_ was added as an internal standard (20 ng/L). The amount of adsorbed geosmin or MIB on GO was calculated by Equation (1):

(1) $$q_{t}=\frac{\left(C_{0}{-C}_{t}\right)V}{m},$$

where *q_t_* is the amount of adsorbed geosmin or MIB (ng/mg) at time *t*, *C*_0_ is the initial concentration of geosmin or MIB (ng/L), *C_t_* is the concentration of geosmin or MIB (ng/L) after time *t*, *V* is the solution volume (L), and *m* is the adsorbent (i.e., GO) mass (mg).

In the adsorption equilibrium experiments, 20 mL aliquots of water samples were added into 28-mL vials, after which the GO dispersion was added (100–500 mg/L) and the vials were mechanically stirred at room temperature. Control samples without GO were also prepared. After 1 h of stirring, 10 mL aliquots of the solution were collected from the vials, and the concentrations of geosmin and MIB were determined using the same procedure described above. The obtained geosmin and MIB adsorption data were then fitted to the Brunauer–Emmett–Teller (BET), Langmuir, and Freundlich isotherm models using Equations (2)–(4) [18,19]:

(2) $$q_{e}=\frac{q_{m}k_{b}C_{e}}{{(C}_{s}{-C}_{e})\left[{1+(k}_{b}-1)\frac{C_{e}}{C_{s}}\right]},$$

(3) $$q_{e}=\frac{Q_{m}bC_{e}}{1+bC_{e}},$$

and

(4) $$q_{e}=KC_{e}{}^{1/n},$$

where *q_e_* is the equilibrium adsorption amount of geosmin or MIB on GO (ng/mg), *C_e_* is the equilibrium concentration of geosmin or MIB (ng/L), *C_s_* is the saturation concentration of geosmin or MIB in water (i.e., water solubility; ng/L), *Q_m_* is the maximum adsorption capacity (ng/mg), *K* is a constant related to the adsorption capacity, and *b* and *n* are constants related to the energy of adsorption. The parameters *q_m_* and *k_b_* are constants related to the monolayer adsorption capacity (ng/mg) and the adsorption energy, respectively. All batch adsorption experiments were conducted in at least duplicates.

## 3. Results

### 3.1. Adsorption Kinetics

**Figure 1:**
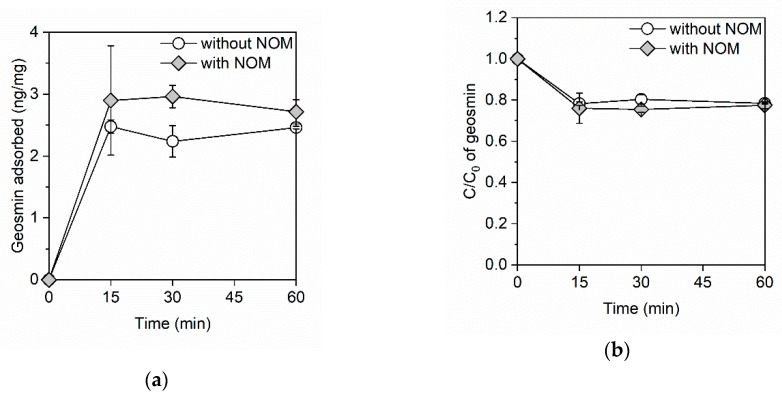
(**a**) The amount of adsorbed geosmin and (**b**) the ratio of residual geosmin on the graphene oxide (GO) in the absence and presence of natural organic matter (NOM). The GO and NOM concentrations were 100 mg/L and 3.6 mg C/L, respectively. The initial concentration of geosmin was ~1 μg/L and the initial pH was between 7.4 and 7.5.

Figure 1a shows the amount of geosmin adsorbed onto the GO in the adsorption kinetics study. In the absence of NOM, the amount of adsorbed geosmin reached 2.5 ng/mg in 15 min, after which no changes were observed, indicating that the adsorption equilibrium of geosmin on the GO was achieved within 15 min. There was no diffusion of the adsorption of geosmin onto the GO when the geosmin reached the adsorption site because of the open-layered structure of GO, which resulted in the rapid adsorption. The surface area of the GO was estimated at 478 m^2^/g according to a previously reported method (Figure S1) [20]. In the presence of NOM, adsorption equilibrium was achieved within 15 min. Figure 1b shows the ratio of residual geosmin. Natural organic matter did not affect geosmin removal, with 22% removal being observed in the absence and presence of NOM. Moreover, the GO exhibited rapid adsorption kinetics for geosmin in the absence and presence of NOM, with the adsorption equilibrium being achieved within 15 min. 

**Figure 2:**
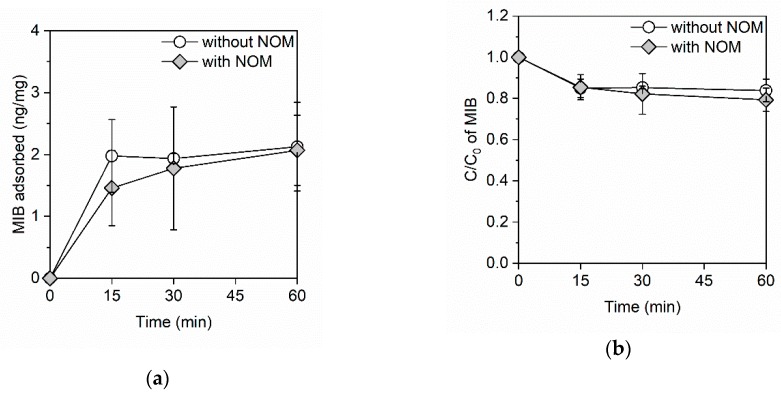
(**a**) The amount of adsorbed MIB and (**b**) the residual ratio of MIB on the GO in the absence and presence of NOM. The GO and NOM concentrations were 100 mg/L and 3.5 mg C/L, respectively. The initial concentration of MIB was ~1 μg/L and the initial pH was 7.4.

Figure 2a shows the amount of MIB adsorbed onto the GO in the adsorption kinetics study. In the absence of NOM, the adsorption equilibrium of MIB was achieved within 15 min and the amount of adsorbed MIB reached 2.0 ng/mg. Conversely, in the presence of NOM, the amount of adsorbed MIB reached 1.5 ng/mg in 15 min and it continued to gradually increase after 15 min. Then, the amount of adsorbed MIB reached 2.1 ng/mg in 60 min. This result indicates that NOM might delay the adsorption of MIB onto the GO. Figure 2b shows the residual ratio of MIB. In the absence of NOM, 15% of the MIB was removed within 15 min, while in the presence of NOM the residual ratio of MIB gradually decreased and 21% of the MIB was removed within 60 min. 

We tried to obtain the kinetic parameters according to the pseudo-first-order and pseudo-second-order kinetic models [19]. However, we could not obtain the parameters because the adsorption capacity of the GO for odor compounds was almost saturated in 15 min.

### 3.2. Adsorption Isotherms

**Figure 3:**
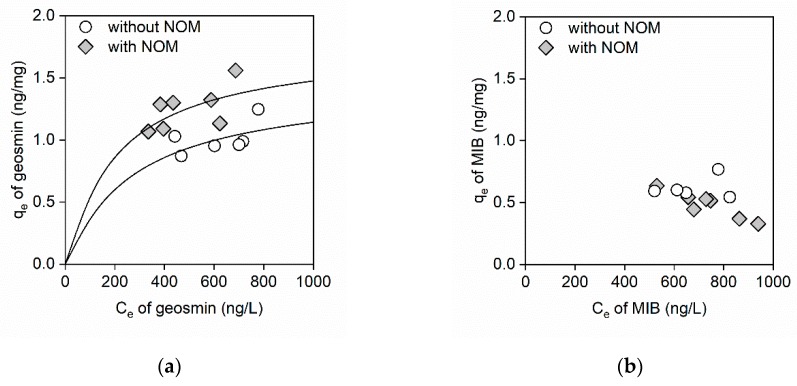
(**a**) Geosmin adsorption isotherms and (**b**) MIB adsorption isotherms on GO in the absence and presence of NOM. The range of GO concentrations was 100–500 mg/L, while the NOM concentration was 2.3 mg C/L, the initial concentrations of geosmin and MIB were ~1 μg/L, and the initial pH was between 7.3 and 7.5. The solid lines are predictions calculated using the Langmuir adsorption isotherm model.

Adsorption equilibrium experiments were conducted for 60 min, which was sufficient to achieve adsorption equilibrium according to the kinetics studies. Figure 3 shows the adsorption isotherms of geosmin and MIB on the GO in the absence and presence of NOM. As shown in Figure 3a, the total amount of adsorbed geosmin increased in the presence of NOM. These results imply that NOM may increase the adsorption sites of GO. A previous study reported that negatively charged NOM increased the dispersibility of GO because of electrical repulsion, which increased the adsorption capacity [21,22]. Conversely, the adsorption isotherm of MIB in the presence of NOM was unusual (Figure 3b), which indicated an interfering effect of NOM on MIB adsorption. These results corresponded with those of a kinetics study of MIB and indicated that the interference occurred because of competition for adsorption sites by MIB and NOM on the GO.

The BET, Langmuir, and Freundlich isotherm models were used to fit the adsorption data [18,19]. Table 2 shows the obtained parameters of the BET or Langmuir isotherm models. Compared to the BET and Langmuir isotherm models, the Freundlich isotherm model did not fit the data well, due to poor correlation (Figure S2). As shown in Table 2, the r^2^ value in the BET model is equal to that in the Langmuir model. Furthermore, the *q_m_* value in the BET model is equal to the *Q_m_* value in the Langmuir model. This result implies the monolayer adsorption of odor compounds on GO, because the BET and Langmuir models assume multilayer adsorption and monolayer adsorption, respectively. The *Q_m_* and *b* of geosmin were 1.46 ng/mg and 3.6 × 10^−3^, respectively. In the presence of NOM, the *Q_m_* of geosmin increased from 1.46 ng/mg to 1.78 ng/mg, and the *b* value decreased from 3.6 × 10^−3^ to 4.9 × 10^−3^. Compared to the r^2^ values obtained from the geosmin adsorption data, the r^2^ value obtained from the MIB adsorption data (0.58) was low.

## 4. Discussion

During the adsorption of geosmin and MIB onto GO, hydrogen bonding, hydrophobic forces, and van der Waals interactions may contribute to the overall interaction between GO and each odor compound. A previous study reported that hydrogen bonding interactions can have a strong effect on the adsorption of phenanthrene and trichloroethylene at neutral pH because of water cluster formation around the oxygen-containing functional groups on GO [22]. In this study, hydrogen bonding interaction may have partially contributed to the adsorption of geosmin and MIB onto the GO because both compounds have one hydroxyl group each on their chemical structure and we conducted the adsorption experiments at a neutral pH (7.3–7.5). The hydroxyl groups of geosmin and MIB may interact with oxygen-containing groups on GO through hydrogen bonding. However, we thought that hydrophobic interaction was dominant because the amount of geosmin adsorbed onto the GO was slightly higher in both kinetics and equilibrium studies compared to the amounts of MIB. These results may be because of the difference in their hydrophobicity. Geosmin has a higher log K_ow_ of 3.70 and is more hydrophobic than MIB (log K_ow_ = 3.13) (Table 1). In addition, the planarity of geosmin might also contribute to the higher adsorbed amount. When compared to geosmin, MIB has less planarity and there might be an effect of steric hindrance in the adsorption of MIB onto GO. A previous study also reported the effects of planarity on the adsorption of GO using planar phenanthrene and nonplanar biphenyl [21]. The difference in the interfering effect of NOM for the adsorption of geosmin and MIB onto GO may also be explained by their hydrophobicity. Indeed, a previous study also showed that the adsorption of hydrophobic phenanthrene (log K_ow_ = 4.68) onto GO was less affected by NOM than hydrophilic trichloroethylene (log K_ow_ = 2.42) [22]. 

A previous study showed that the adsorption capacity of PAC for geosmin and MIB ranged from 90 to 2000 ng/mg at an equilibrium concentration of 100 ng/L [5]. When compared to those values, the adsorption capacity of GO obtained in this study was 2–3 orders of magnitude lower, which may have been because of the lower specific surface area and higher surface hydrophilicity compared to PAC. Considering the high cost of GO and its low adsorption capacity for odor compounds, this method cannot currently be applied to actual water treatment. The oxygen-containing functional groups on GO can increase the dispersion of GO in water; however, they also increase the surface hydrophilicity, which weakens the hydrophobic interaction between GO and odor compounds. Previous studies of SPAC also reported the decreased adsorption capacity due to milling to the submicron particle size because normal-milled SPACs have more oxygen/hydrogen-containing functional groups and less hydrophobicity [23,24]. 

This study has some limitations. First, we used synthetic water and only investigated the effects of NOM on the adsorption of geosmin and MIB on GO. Second, we used commercially available GO and did not characterize it. Therefore, further research about the effects of other parameters (e.g., GO properties, ionic strength, and competing ions) are needed.

## 5. Conclusions

Graphene oxide exhibited fast adsorption kinetics for geosmin and MIB because of its open-layered structure. Adsorption equilibrium was achieved within 15 min of contact. The reaction of graphene oxide with odor compounds likely occurs mainly through hydrophobic interactions. Therefore, the capacity of GO to adsorb geosmin was larger than that for MIB because geosmin has greater hydrophobicity. The NOM likely interfered with the adsorption of MIB by the GO via competition for adsorption sites. However, NOM did not affect the adsorption kinetics of the GO for geosmin, and NOM likely increased the amount of adsorbed geosmin via an increase in the GO adsorption sites. These results may also reflect differences in the hydrophobicity of geosmin and MIB. Although the GO showed fast adsorption kinetics for geosmin and MIB, the adsorption capacity of the GO for both odor compounds was substantially lower because of the higher surface hydrophilicity of GO. Controlling the content of oxygen-containing functional groups on GO may improve the capacity for the adsorption of both odor compounds.

## Figures and Tables

**Table 1:** Chemical structure, molecular weight, octanol–water partition coefficient (log K_ow_), and water solubility at 20 °C (C_s_) of geosmin and 2-methylisoborneol (MIB).

Compound Name	Chemical Structure	Molecular Weight (g/mol)	log K_ow_^1^	C_s_^1^ (mg/L)
Geosmin	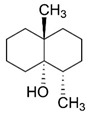	182.3	3.70	150.2
MIB	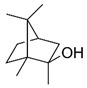	168.3	3.13	194.5

^1^ These values were obtained from a previous study [17].

**Table 2:** *q_m_*, *k_b_*, *Q_m_*, *b*, and r^2^ values in the Brunauer–Emmett–Teller (BET) and Langmuir isotherm models.

Model	Adsorbate	*q_m_* (ng/mg)	*k_b_*	*Q_m_* (ng/mg)	*b*	r^2^
BET	Geosmin	1.46	5.4 × 10^5^	-	-	0.69
	Geosmin–NOM	1.78	7.4 × 10^5^	-	-	0.73
	MIB	0.67	3.0 × 10^5^	-	-	0.58
	MIB–NOM	-	-	-	-	-
Langmuir	Geosmin	-	-	1.46	3.6 × 10^−3^	0.69
	Geosmin–NOM	-	-	1.78	4.9 × 10^−3^	0.73
	MIB	-	-	0.67	1.5 × 10^−2^	0.58
	MIB–NOM	-	-	-	-	-

## Supplementary Materials

The following are available online at https://www.mdpi.com/1660-4601/16/11/1907/s1, Figure S1: Plot of the amount of methylene blue necessary to reach the maximum absorption intensity of the 580 nm band versus increasing concentrations of GO., Figure S2: Plot of log*Ce* value versus log*qe* value to obtain the parameter *K* and *n* in the Freundlich adsorption isotherm model.
