# Supplementary material for: Application of Graphene Oxide for Adsorption Removal of Geosmin and 2-Methylisoborneol in the Presence of Natural Organic Matter
Source: Int J Environ Res Public Health. 2019 May 30;16(11):1907. doi: 10.3390/ijerph16111907 (PMC6603623; doi:10.3390/ijerph16111907)
Supplement: Supplementary file 1 [file ijerph-16-01907-s001.pdf]

Supplementary Materials

Application of Graphene Oxide for Adsorption Removal of Geosmin and 2-Methylisoborneol in the Presence of Natural Organic Matter

Akira Hafuka [1,*], Takahiro Nagasato [2] and Hiroshi Yamamura [2]

[1] Center for Regional Environmental Research, National Institute for Environmental Studies (NIES), 16-2 Onogawa, Tsukuba, Ibaraki 305-8506, Japan

[2] Department of Integrated Science and Engineering for Sustainable Society, Faculty of Science and Engineering, Chuo University, 1-13-27 Kasuga, Bunkyo-ku, Tokyo 112-8551, Japan; tn060613@gmail.com (T.N.), yamamura.10x@g.chuo-u.ac.jp (H.Y.)

* Correspondence: hafuka.akira@nies.go.jp; Tel.: +81-29-886-0973

Received: 7 May 2019; Accepted: 28 May 2019; Published: 30 May 2019

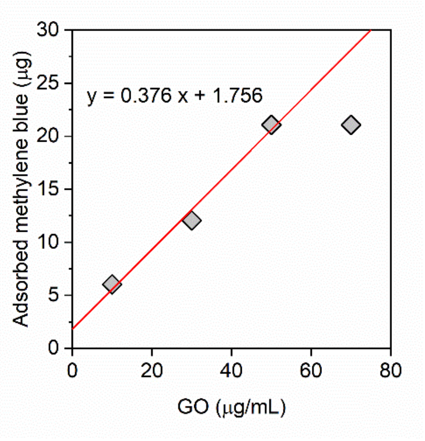

Figure S1. Plot of the amount of methylene blue necessary to reach the maximum absorption intensity of the 580 nm band versus increasing concentrations of GO. The volume of GO dispersion was 2 mL. Surface area of GO in water was measured according to a previously reported method [20]. Absorption spectra were measured using a UV-Vis spectrophotometer (UV-1800; Shimadzu Corporation, Kyoto, Japan). Spectroscopic measurements were conducted by adding 2 mL of each GO dispersion (10-70 μg/mL) to quartz cells with a cross section of 1 cm × 1 cm, and small aliquots of a stock solution of methylene blue were added up to 200 μL. Figure S1 shows the amount of adsorbed methylene blue on GO. Considering 2.54 m^2 as the area covered by mg of methylene blue [20], a surface area of GO was estimated at 478 m^2/g from the slope of the plot (i.e., 0.376 μg/(μg/mL) ÷ 2 mL × 2.54 m^2/mg = 0.477 m^2/mg) .

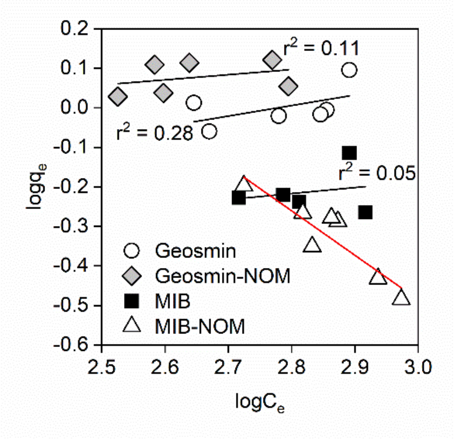

Figure S2. Plot of $logC_e$ value versus $logq_e$ value to obtain the parameter K and n in the Freundlich adsorption isotherm model.
